# Pericardial Inflammatory Mediators in Patients Undergoing Cardiac Surgery: Towards a Biomarker-Informed Clinical Practice

**DOI:** 10.31083/RCM49311

**Published:** 2026-04-02

**Authors:** Michael Blackledge, Hamza Nasir Chatha, Angel Luis Fernández González, Yasir Abu-Omar, Adham Elsherbini, Mohammad El Diasty

**Affiliations:** ^1^School of Medicine, Case Western Reserve University, Cleveland, OH 44106, USA; ^2^Department of Surgery, University Hospitals Cleveland Medical Center, Cleveland, OH 44106, USA; ^3^Division of Cardiovascular Surgery, University Hospital, 15706 Santiago de Compostela, Spain; ^4^Division of Cardiac Surgery, Harrington Heart and Vascular Institute, University Hospitals Cleveland Medical Center, Cleveland, OH 44106, USA; ^5^Faculty of Medicine, University of Toronto, Toronto, ON M5S 1A8, Canada

**Keywords:** pericardium, pericardial fluid, inflammatory markers

## Abstract

Postoperative atrial fibrillation, heart failure, and pericardial effusion remain frequent complications after cardiac surgery. Pericardial fluid is a localized inflammatory compartment in close contact with the myocardium and may provide information that complements systemic biomarkers. This narrative review summarizes inflammatory mediators identified in pericardial fluid after cardiac surgery and their associations with postoperative outcomes. We discuss potential mechanistic links between pericardial inflammation and pericardial fluid markers, as well as practical limitations related to sampling, timing, and standardization. Although pericardial fluid biomarkers may augment existing clinical risk models, prospective studies are needed to determine their incremental value and clarify their role in perioperative management.

## 1. Introduction

The pericardium is a two-layered, fibroserous sac that contributes to the mechanical protection and stability of the heart, as well as neurohumoral and immunological regulation. The space between the visceral and parietal layers of the serous pericardium contains the pericardial fluid (PCF). During pathological conditions, the pericardial space becomes a site of ‘dynamic inflammatory activity’ with several biochemical and molecular changes. Surgical trauma, myocardial disorders, and inflammatory changes can provoke an intense inflammatory response in the pericardial space, leading to cellular and molecular changes and the selective accumulation of inflammatory mediators in the pericardial compartment. Multiple studies have reported the role of local pericardial inflammation in the pathogenesis of adverse clinical outcomes in patients undergoing cardiac surgery. However, the utilization of these biomarkers in routine clinical practice remains very limited. This may be attributed, in part, to technical challenges in collecting and processing pericardial fluid samples, as well as the scarcity of solid evidence on the predictive value of these biomarkers in the clinical setting. This review aims to consolidate current evidence and provide an overview of pericardial inflammatory mediators and their potential role in predicting clinical outcomes in patients undergoing cardiac surgery.

## 2. Methods

This narrative review synthesizes current evidence on inflammatory mediators in pericardial fluid and their clinical relevance in cardiac surgery. Literature searches covered PubMed and the Cochrane Library for English-language, peer-reviewed articles published through February 2025, using combined search terms such as pericardial fluid, inflammation, cardiac surgery, cytokines, natriuretic peptides, galectin-3, troponin, and microRNAs. Both experimental, translational, and clinical studies were included in the review. Given the heterogeneity in study designs and outcomes, a systematic review or meta-analysis was not feasible; therefore, findings are summarized narratively, with particular emphasis on limitations and clinical relevance.

### 2.1 Characteristics of the Pericardium and Pericardial Fluid

Pericardial fluid (PCF) is mainly an ultrafiltrate of plasma. It is derived from the visceral pericardial microvasculature [1]. This fluid originates in the cardiac interstitium and diffuses across the epicardial layer into the pericardial sac. The composition of PCF can represent the biochemical and hormonal microenvironment of the cardiac interstitium. The pericardial sac also encloses epicardial adipose tissue. This tissue is important for biochemical, hormonal, and metabolic regulation of cardiac homeostasis [2]. PCF has a low turnover rate. This is shown by extended residence times of radiolabeled growth factors and albumin in animal models. Therefore, PCF has emerged as an important marker for the diagnosis and prognosis of different cardiac disorders, such as myocardial ischemia, congestive heart failure, cardiac arrhythmias, and pathological pericardial effusions.

The pericardium can respond to injury in two ways: fluid exudation leading to PCF accumulation, or acute inflammation. Pericardial disorders may involve one main component, but both reactions often occur together [3]. Clinical presentation varies from subtle changes to life-threatening cardiac tamponade due to PCF accumulation and impaired cardiac output. These well-known features of the pericardium matter postoperatively because the pericardial space acts as a biologically active compartment, where inflammatory mediators can persist near the atrial and ventricular myocardium.

### 2.2 Pericardial Inflammation After Cardiac Surgery

Cardiac surgery triggers a systemic inflammatory response that contributes substantially to postoperative organ dysfunction and coagulation disorders that can adversely impact clinical outcomes [4, 5, 6].

**Fig. 1.**
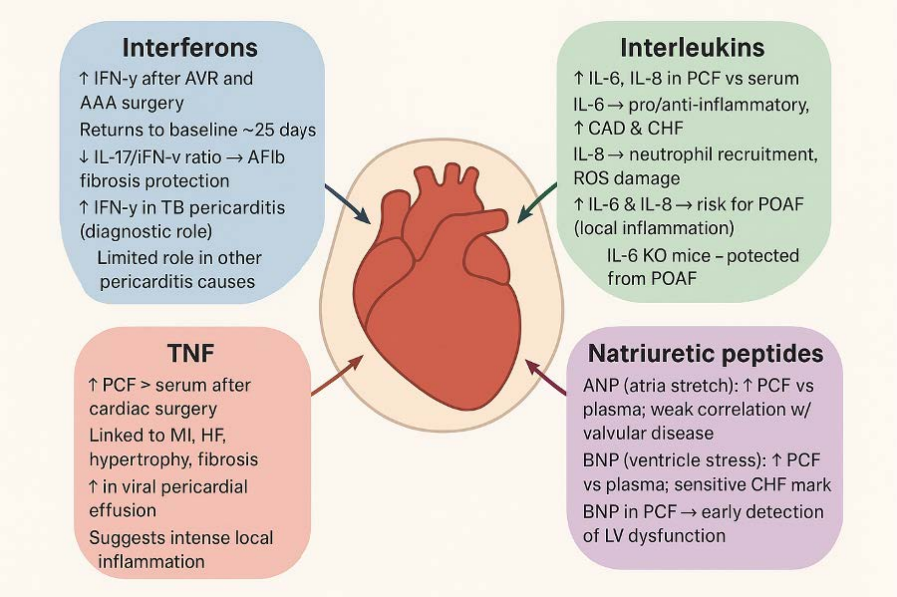
**Biomarkers in the pericardial fluid**. IFN, Interferon; AVR, Aortic Valve Replacement; AAA, Abdominal Aortic Aneurysm; PCF, Pericardial Fluid; CAD, Coronary Artery Disease; CHF, Congestive Heart Failure; ROS, Reactive Oxygen Species; POAF, Postoperative atrial fibrillation; KO, Knock-out; MI, Myocardial Infarction; HF, Heart Failure; ANP, Atrial Natriuretic Peptide; BNP, B-type Natriuretic Peptide; LV, Left Ventricle.

In the perioperative period of cardiac surgery, inflammatory markers display a temporal pattern that reflects the sum of baseline patient risk profile, the extent of surgical trauma, Cardiopulmonary bypass (CPB)-induced systemic inflammation, and postoperative organ dysfunction. At baseline, patients undergoing cardiac surgery often present with elevated markers such as C-reactive protein (CRP) and interleukin-6 (IL-6) secondary to underlying cardiovascular disease, complex comorbidities (e.g., congestive heart failure, coronary artery disease), and chronic low-grade inflammation. Following surgery, an early increase in acute-phase reactants and cytokines is consistently reported; serum CRP typically peaks at ~48 hours postoperatively, while cytokines such as IL-6 and IL-8 show a rapid surge within the first 24 h. Several recent investigations have also shown that the pericardial fluid compartment undergoes a more pronounced inflammatory response than plasma in the early postoperative period, with marked elevations of IL-6, IL-8, tumor necrosis factor-α (TNF-α), and other mediators, thus highlighting the concept of compartmentalization of the inflammatory process within the pericardial cavity in these patients [7, 8]. The biomarkers discussed below are depicted in Fig. 1.

### 2.3 Pericardial Inflammatory Markers in Clinical Practice 

Pericardial inflammation can lead to several changes in the biochemical composition of pericardial fluid and serum. While serum markers such as CRP and erythrocyte sedimentation rate (ESR) are widely used in the management of pericardial inflammation, these represent systemic inflammation and lack specificity for pericardial processes. Furthermore, PCF contains a concentrated proportion of acute-phase reactants, cytokines, and pro-inflammatory mediators, which are upregulated during an inflammatory process. These may be useful in differentiating between infectious and non-infectious etiologies, in addition to their role as prognostic markers. Despite limited use in clinical practice, some of the most important ones are discussed below [7, 8]. These biomarkers are summarized in Table 1.

**Table 1.** **Overview of proposed biomarkers and current clinical applicability**.

Marker	Marker class	Key findings in pericardial fluid	Proposed clinical application/significance	Limitations	Timing and collection method
Interferon-γ (IFN-γ)	Cytokine (Interferon)	Elevated PCF levels vs serum in cardiac surgery; high sensitivity and specificity for tuberculosis pericarditis; no PCF–serum gradient in autoimmune, neoplastic, or viral pericarditis	Potential diagnostic marker for tuberculous pericarditis; possible association with reduced atrial fibrosis and atrial fibrillation	Limited utility outside TB pericarditis; largely correlational data; inconsistent findings across etiologies	Intraoperative Collection
Interleukin-6 (IL-6)	Cytokine (Interleukin)	Markedly higher PCF vs serum levels; elevated in chronic heart failure; increased following cardiac surgery	Indicator of localized pericardial inflammation; potential predictor of postoperative atrial fibrillation (POAF)	Not disease-specific, reflective of inflammation but not etiology	Intraoperative Collection
Interleukin-8 (IL-8)	Cytokine (Interleukin)	Significantly higher levels of IL-8 in PCF vs serum	Marker of neutrophil-driven local inflammation; potential predictor of POAF	Limited prospective data; influenced by surgical trauma	Intraoperative Collection
Tumor Necrosis Factor (TNF)	Cytokine	Higher PCF than serum in cardiac surgery; elevated in viral pericardial effusions	Marker of intense local inflammatory response; potential indicator of viral pericarditis	Overlaps with other inflammatory conditions; unclear prognostic value	Intraoperative Collection
Atrial Natriuretic Peptide (ANP)	Natriuretic peptide	Higher PCF than plasma in cardiovascular surgery; weak correlation with valvular disease	Possible marker of cardiac wall stress or valvular pathology	Sparse and outdated literature; limited clinical validation	Intraoperative collection
B-type Natriuretic Peptide (BNP)	Natriuretic peptide	PCF concentrations up to 22-fold higher than plasma in LV dysfunction; correlates with CHF and AF	Potential early marker of ventricular dysfunction and heart failure	Serum BNP already well-validated marker of disease	Intraoperative collection
Galectin-3 (Gal-3)	Lectin	Experimental models show increased fibrosis when present in pericardial space	Potential biomarker and mediator of cardiac fibrosis and remodeling	Primarily preclinical evidence; not yet clinically validated	Intraoperative Collection
MicroRNAs (miRNAs)	Non-coding RNA	Distinct PCF miRNA profile; altered expression in STEMI; some miRNAs linked to HF and inflammation	Emerging diagnostic and therapeutic targets; possible paracrine modulators	Underdeveloped field; heterogeneous findings; unclear clinical thresholds	Intraoperative Collection
Troponin I (cTnI)	Myocardial injury marker	Elevated PCF levels post-cardiac surgery; higher levels associated with POAF	Possible marker of perioperative myocardial injury and POAF risk	Serum cardiac troponin is already well-validated marker of ischemia;	Postoperative Collection via Pericardial Drain
C-reactive Protein (CRP)	Acute-phase protein	Detectable in PCF despite size; reflects local inflammation	Marker of pericardial inflammation and disease activity	Poor specificity; limited etiologic discrimination	Intraoperative Collection

### 2.4 Clinically Actionable Biomarkers

#### 2.4.1 Natriuretic Peptides

The primary functions of Atrial natriuretic peptide (ANP) include natriuresis, diuresis, and vasodilation, thus controlling blood pressure [9]. The study by Soós *et al*. [10] found that, in patients undergoing cardiovascular surgery, pericardial ANP levels were significantly higher than venous plasma concentrations. Additionally, they found a weak correlation between PCF ANP concentrations and valvular heart disease [10]. However, research on ANP in pericardial fluid is scarce, with most studies published over 20 years ago. This raises concerns about the use of pericardial ANP in practice without further research.

B-type natriuretic peptide (BNP) promotes natriuresis, diuresis, and vasodilation and inhibits the renin-angiotensin-aldosterone system during periods of increased ventricular wall stress [11, 12]. In patients undergoing cardiac surgery with impaired left ventricular function, pericardial BNP levels were 22-fold higher than plasma concentrations (*p* < 0.005) [13]. For patients presenting with dyspnea, rapid BNP measurement is a highly specific (0.76) and sensitive (0.90) marker for congestive heart failure [14]. Due to the tendency of BNP to be much higher in the PCF than in plasma, PCF BNP levels may be useful in the early detection of ventricular dysfunction and heart failure. In a recent review of literature, El-Sherbini *et al*. [11] reported that pericardial fluid levels of natriuretic peptides correlated with some cardiac disorders, such as congestive heart failure and atrial fibrillation.

#### 2.4.2 Interleukins

Interleukins modulate immune response as well as cell proliferation and differentiation. IL-6 concentrations were significantly increased in the PCF (183.81 ± 94.49 pg/mL) compared to serum levels (11.49 ± 6.08 pg/mL, *p* < 0.01) [15]. This was also reported for IL-8, with PCF levels (28.12 ± 20.92 pg/mL) being significantly higher than serum levels (7.82 ± 5.69 pg/mL; *p* < 0.01) [16]. This can suggest local production of cytokines that are either synthesized within (from the epithelial cells) or secreted (from the epicardial adipose tissue) into the pericardial cavity. IL-6 is known to increase in patients receiving elective cardiac surgery [17]. IL-6 has also been associated with higher concentrations in the PCF in patients with chronic heart failure when compared with patients without heart failure (749.60 ± 468.06 vs. 303.45 ± 275.00 pg/mL; *p* < 0.05) [15]. Mice with an IL-6 Knockout (KO) mutation were completely protected with significantly fewer IL-6 KO mice developing sustained, spontaneous postoperative atrial fibrillation (ssPOAF) (0/10 affected) and non-sustained spontaneous (nsPOAF) (1/10 affected), when compared to age and weight-matched wildtype mice (4/10 developed ssPOAF, *p* = 0.01; 5/10 developed nsPOAF, *p* = 0.044) in the 7 days following surgery [18]. In a retrospective study, coronary artery bypass grafting (CABG) patients who developed postoperative atrial fibrillation (POAF) had significantly higher 2-hour postoperative IL-8 concentrations (*p* < 0.05) than those who did not [19]. Elevated pericardial IL-6 and IL-8 levels, compared to serum, demonstrate a robust, compartmentalized local inflammatory response that potentially can be a useful clinical indicator of POAF.

### 2.5 Markers With Limited Evidence

#### 2.5.1 Interferons

Interferons are cell-signaling glycoproteins that are typically released in association with the innate immune system’s response to infection and neoplastic proliferation. In patients receiving aortic valve replacement surgery, there was a significantly increased interferon gamma (IFN-γ) concentration relative to basal concentrations (PCF: 5.34 ± 5.12 pg/mL; Serum: 0.83 ± 0.65 pg/mL; *p* < 0.01) [16]. Serum IFN-γ levels were also significantly increased in patients undergoing abdominal aortic aneurysm repair compared with preoperative levels, remaining elevated up to 9 days after surgery and returning to normal levels by 25 days (*p* < 0.001) [20]. A low IL-17/IFN-γ ratio is negatively associated with areas of decreased electrical activity in patients with diagnosed atrial fibrillation, possibly suggesting that increased IFN-γ and decreased IL-17 levels may have a protective role against fibrotic changes that lead to atrial fibrillation [7]. Elevated INF-γ levels had high sensitivity (0.97) and specificity (0.99) for the diagnosis of tuberculosis pericarditis [21]. However, in pericarditis with autoimmune, neoplastic, or viral etiologies, there was no significant difference in INF-γ between PCF and serum [22]. Due to this, there appears to be limited clinical usage of pericardial INF-γ levels for diagnosing tuberculosis-related pericarditis; there are no concrete findings and only a correlation to the development of POAF.

#### 2.5.2 Tumor Necrosis Factor

Tumor necrosis factor (TNF) functions to promote leukocyte recruitment, stimulation of acute phase reactants, and mediation of cell death. Increased serum TNF has been associated with many forms of cardiac pathology, including myocardial infarction, heart failure, hypertrophy, and fibrosis [23]. In patients receiving cardiac surgery, the PCF levels of TNF were significantly higher than those of the serum (Serum: 3.18 ± 1.06 pg/mL; PCF: 4.271 ± 0.28 pg/mL; *p* < 0.05) [16]. In the context of pericarditis, pericardial TNF levels were significantly higher in virus-related pericardial effusion than in neoplastic, autoreactive, lymphocytic, or iatrogenic etiologies [22]. Like IL-6 and IL-8, elevated TNF levels may indicate severe local inflammation, and further research is needed to determine whether TNF concentration in PFT can serve as an indicator of surgical morbidity and mortality.

#### 2.5.3 C-Reactive Protein (CRP)

C-reactive protein (CRP) is a relatively large protein at 118 kDa, which cannot easily diffuse into the pericardial sac from the epicardium, which is typically permeable for molecules smaller than 40 kDa [24]. Despite this, CRP has been detected within the pericardial fluid of CABG patients who were receiving surgery for stable angina, unstable angina, and surgery following myocardial infarction [24]. This is likely due to increased vascular permeability caused by inflammatory changes local to the pericardium and surrounding tissues. CRP has also been proposed for monitoring acute pericarditis to assess disease severity and treatment response [25]. While CRP in the PCF can confirm the presence of local inflammation, it does not reliably distinguish between causes of disease or inflammation.

### 2.6 Experimental Markers

#### 2.6.1 Galectin-3

Galectin-3 (Gal-3) is a β-galactoside-binding lectin secreted by activated macrophages and other immune cells in response to tissue injury. It plays a central role in mediating fibrotic remodeling and chronic inflammation in cardiovascular disease [26]. Gal-3 has been proposed as a potential biomarker for heart disease because Gal-3-expressing macrophages are present in the early stages of cardiac fibrosis and remodeling [27]. When galectin-3 is transfused into the pericardial sac of rats, there is a significant increase in collagen production and cell proliferation, eventually leading to the development of heart failure [28]. These studies are preliminary for clinical application and indicate that pericardial Gal-3 is a potential subject for future research.

#### 2.6.2 MicroRNAs

MicroRNAs (miRNAs) in PCF act as paracrine signaling molecules that modulate gene expression in the nearby cardiac cells. PCF contains a distinct miRNA profile as compared to the heart. For example, miR-1, which is abundant in cardiac tissues, had very low levels in PCF and was detectable in only 28/51 samples [29]. There has been limited research on the significance of miRNAs already present in the PCF; however, some miRNAs have previously been identified as possible diagnostic biomarkers for heart failure [30]. Delivery of miRNAs to the pericardial sac has been proposed as a therapeutic approach for cardiovascular disease [31]. Several miRNAs have been shown to be upregulated in PCF in patients with ST-Elevation Myocardial Infarction (STEMI), and miR-22-3p levels are significantly higher in STEMI patients than in Non–ST-Elevation Myocardial Infarction (NSTEMI) patients or non-ischemic controls [32]. The study of miRNAs in PCF remains underdeveloped, and further research is needed to determine their clinical significance.

#### 2.6.3 Troponins

Within the PCF, there is evidence that there is increased troponin I (cTnI) in the PCF following cardiac surgery, likely due to myocardial damage during surgery. Butts *et al*. [3] found that cTnI levels in the PF were significantly increased 48 hours postoperatively in individuals who developed POAF (0.22 ± 0.05 µg/mL) compared with those who did not (0.09 ± 0.07 µg/mL; t = 3.125; *p* = 0.01). There are downsides, however: studies comparing cTnI in the PCF and serum have found that PCF cTnI measurements were less useful for diagnosing myocardial ischemia after CABG [33, 34]. PCF is also difficult to obtain preoperatively, and postoperatively, it can be harvested only from pericardial drains, which are often removed within 24 hours [11, 33]. Troponins show some evidence of being useful biomarkers in PCF; more research is needed to draw definitive conclusions.

## 3. Limitations

Despite growing interest in pericardial fluid biomarkers, several important limitations must be acknowledged. First, most available evidence is derived from small, single-center, observational or translational studies, often with heterogeneous patient populations, surgical procedures, and sampling protocols. Differences in the timing of pericardial fluid collection (intraoperative vs. early or late postoperative), variability in drain-based sampling, and lack of standardized processing methods limit direct comparison across studies. Second, many associations between pericardial biomarkers and postoperative outcomes are correlational, and causality cannot be inferred in most clinical settings. While experimental data support mechanistic roles for certain mediators, such as IL-6, in fibrosis and electrical remodeling in humans, these biomarkers may primarily reflect underlying myocardial stress and inflammatory burden rather than act as independent drivers of pathology. Third, validated cutoff values, assay standardization, and reproducibility across laboratories are largely lacking, which currently restricts clinical implementation. Practical considerations, including the feasibility of routine pericardial fluid sampling, narrow postoperative time windows before drain removal, inter-institutional variability, and cost-effectiveness, further challenge real-world adoption. Finally, although pericardial fluid biomarkers may provide biologically enriched information compared with systemic markers, robust prospective studies demonstrating incremental predictive value beyond existing clinical risk scores and serum biomarkers are still needed.

## 4. The Need for Biomarkers-Informed Clinical Practice

Despite major advances, current clinical postoperative risk models rely heavily on preoperative variables (e.g., age, left atrial size, left ventricular function, surgery type, and CPB), intraoperative metrics (e.g., procedure type and urgency), and nonspecific systemic markers (e.g., leukocyte count, CRP). While useful, these models capture only a part of the risk spectrum and lack insight into the localized biological processes occurring within the pericardial environment [9]. Subsequently, clinicians often lack timely markers indicative of postoperative complications. Further efforts should be directed toward the PCF, which can provide insight into the dynamic responses of body organs to surgical trauma, thereby enabling early detection of complications [35].

Moreover, it has been well established that systemic biomarkers such as C-reactive protein and troponin are pivotal for clinical assessment, reflecting localized disease activity and myocardial involvement, but are limited in specificity. In the postoperative setting, when systemic inflammation is generalized, localized pericardial fluid biomarkers may provide insight into compartmental changes within the pericardium [36].

Furthermore, the pericardial space is uniquely placed as it holds the epicardium, autonomic fibers, and coronary vasculature. Pericardial cytokines and natriuretic peptides are consistently higher in PCF than in plasma, making PCF an early-phase reporter of myocardial stress, microinjury, and fibrosis [8]. Translational efforts to integrate the markers discussed in this review into perioperative care may allow clinicians to detect subclinical injury before clinical deterioration becomes clear [16]. Specifically, routine measurements of PCF biomarkers may enable proactive arrhythmia prevention, refined hemodynamic monitoring, heart failure management, improved identification of pericardial tamponade or effusion, and enhanced differentiation of superficial versus deep myocardial injury.

Measuring markers pre-operatively and in the immediate post-operative period (e.g., 24–48 h) in interventional randomized studies, via pericardial drain sampling, may help identify the subgroup of patients at higher risk of developing postoperative complications such as heart failure, cardiac tamponade, myocardial ischemia, or POAF, thus allowing for close monitoring and the implementation of early preventative and therapeutic strategies (e.g., the initiation of anti-coagulation or anti-inflammatory regimens, and the early start of anti-arrhythmic medications). Further, it has been demonstrated that adding biomarker-derived data to existing clinical risk assessment scores may improve discrimination and calibration by capturing the individualized biologic response to surgery rather than relying solely on clinical predictors [37]. Finally, understanding the biological and clinical roles of pericardial biomarkers, such as inflammatory cytokines, can enable the identification of novel therapeutic targets and the initiation of targeted therapies to prevent postoperative complications.

## 5. Conclusion

The pericardial fluid is a dynamic, mediator-rich environment that exhibits a compartmentalized, distinctive pattern of inflammation after cardiac surgery. The accumulation of pro-inflammatory and pro-fibrotic biomarkers within the pericardial cavity can play an important role in risk-stratification for patients undergoing cardiac surgery, as well as in the early detection and prevention of major postoperative complications such as atrial fibrillation and heart failure. The addition of biomarker-derived data to existing clinical risk scores can improve their performance, enable the implementation of patient-specific preventive strategies, and potentially lead to improved clinical outcomes.

## Abbreviations

ANP, Atrial Natriuretic Peptide; AF, Atrial Fibrillation; BNP, B-type Natriuretic Peptide; CABG, Coronary Artery Bypass Grafting; CAM, Cellular Adhesion Molecules; CPB, Cardiopulmonary Bypass; CRP, C-Reactive Protein; cTnI, Cardiac Troponin I; ECM, Extracellular Matrix; ESR, Erythrocyte Sedimentation Rate; Gal-3, Galectin-3; HF, Heart Failure; IFN, Interferon; IFN-α, Interferon-alpha; IFN-γ, Interferon-gamma; IL, Interleukin; IL-1β, Interleukin-1 beta; IL-6, Interleukin-6; IL-8, Interleukin-8; KO, Knockout; LV, Left Ventricle/Left Ventricular; miRNA, MicroRNA; NSTEMI, Non–ST-Elevation Myocardial Infarction; PCF, Pericardial Fluid; POAF, Postoperative Atrial Fibrillation; ROS, Reactive Oxygen Species; ssPOAF, Sustained Spontaneous Postoperative Atrial Fibrillation; nsPOAF, Non-sustained Spontaneous Postoperative Atrial Fibrillation; STEMI, ST-Elevation Myocardial Infarction; SIRS, Systemic Inflammatory Response Syndrome; TNF, Tumor Necrosis Factor; TNF-α, Tumor Necrosis Factor-alpha.
